# NIR-Responsive Microbubble Delivery Platforms for Controlled Drug Release in Cancer Therapy

**DOI:** 10.3390/ma18122725

**Published:** 2025-06-10

**Authors:** Kibeom Kim, Been Yoon, Jungmin Lee, Gyuri Kim, Myoung-Hwan Park

**Affiliations:** 1 Department of Convergence Science, Sahmyook University, Seoul 01795, Republic of Korea; kibumsy@syu.ac.kr (K.K.); gyurikim716@gmail.com (G.K.); 2 Department of Chemistry and Life Science, Sahmyook University, Seoul 01795, Republic of Korea; 3 N to B Co., Ltd., Business Incubator Center, Hwarang-ro, Nowon-gu, Seoul 01795, Republic of Korea; beeny0102@gmail.com (B.Y.); jm.lee090394@gmail.com (J.L.)

**Keywords:** cancer therapy, drug delivery system, microbubble, controlled release

## Abstract

Cancer remains one of the leading causes of death worldwide. Therefore, the continuous development of effective therapeutic strategies is necessary. Conventional anticancer chemotherapy has low bioavailability and poor systemic distribution, resulting in serious side effects and limited therapeutic efficacy. To address these limitations, drug delivery systems that respond to external stimuli have been developed to release drugs at specific sites. In this study, a phase transition-based bubble-mediated emulsion system was developed to enable near-infrared (NIR)-induced drug release. This system consists of an oil phase, 2H,3H-perfluoropentane (PFC), a fluorinated liquid gas that evaporates at a certain temperature, and encapsulated IR-780 and paclitaxel to maintain stable microbubbles. Under NIR irradiation, IR-780 exhibits a photothermal conversion effect, which increases the temperature. Above the critical temperature, PFC undergoes a phase transition into gas, forming gas bubbles. This phase transition leads to a rapid volume expansion, destroys the microbubble structure, and triggers drug release. The NIR-responsive microbubble system developed in this study facilitated targeted and selective drug release through precise temperature control using the photothermal effects and phase transition. This system provides a novel platform to improve the efficacy of cancer therapies.

## 1. Introduction

Cancer is one of the leading causes of death worldwide, and there is a continuous demand for the development of effective treatments [1,2,3,4,5]. Conventional cancer chemotherapy causes toxicity in normal tissues owing to low systemic bioavailability and distribution, which leads to side effects and treatment inefficacy [6,7]. To overcome these challenges, drug delivery systems, particularly stimulus-responsive systems, have been developed [8,9,10,11]. These systems can control the release of drugs into specific organs to reduce side effects [12,13,14]. Microbubble (MB)-based systems have attracted considerable attention as stimuli-responsive drug delivery vehicles [15,16,17,18,19].

MB are spherical particles composed of a gas in a liquid medium, and their high surface-to-volume ratio allows the efficient loading of drugs or substances for treatment or for disease diagnosis [20,21,22,23]. In addition, MBs undergo physical changes, such as size deformation and collapse, in response to physical stimuli, such as ultrasound or light, which facilitate controlled drug release [24,25,26,27,28,29]. In particular, MBs transport loaded drugs through blood vessels because of their buoyancy and size characteristics, maximizing therapeutic efficacy by generating local mechanical forces, such as microjets or acoustic cavitation, when the bubbles collapse [30,31,32,33,34,35]. Conventional bubble-based drug delivery systems induce drug release through bubble activation via ultrasound [36,37,38]. Ultrasound, which penetrates deep tissue, facilitates drug release and improves tissue permeability by utilizing the resonance phenomenon with MBs [39,40,41,42]. However, ultrasound-based systems have limitations in that they must match the resonant frequency of the bubbles, and energy can be scattered in certain tissues [43,44,45,46,47]. In addition, precise localized heating is difficult and can damage normal tissues when high energy is required [48,49,50]. To overcome these challenges, optical methods, particularly near-infrared (NIR) light, have emerged as a promising alternative for externally triggered drug delivery. NIR light can be selectively irradiated to specific areas, allowing drug release to be controlled at the desired area, and has excellent tissue penetration, facilitating its application to deep tissues [51,52,53,54,55]. In contrast to ultrasound, NIR does not depend on a specific resonance frequency and allows for more precise temperature control [56,57,58,59,60].

Compared to conventional ultrasound-triggered microbubble systems, which rely on mechanical resonance and often suffer from limited spatial precision and energy attenuation in deep tissues, NIR-based activation provides several advantages. NIR light allows for more precise and localized heating without the need for physical contact or resonance tuning. While previous IR-780-based systems have primarily focused on thermal ablation or surface heating, the present system incorporates IR-780 with a volatile fluorinated liquid-gas, resulting in a phase transition-driven collapse mechanism for controlled drug release. This dual-mode strategy, which integrates photothermal activation with gas-phase expansion, represents a significant departure from previously reported single-mode delivery platforms.

**Scheme 1:**
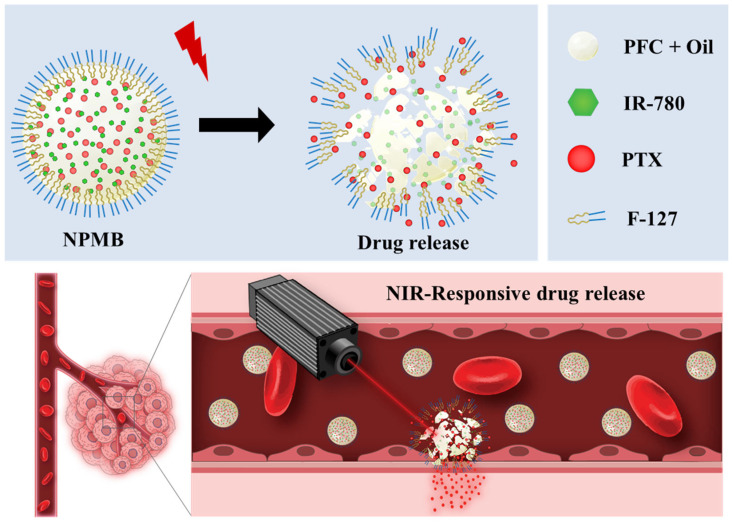
Illustration of the NPMB system enabling controlled drug release for targeted cancer therapy.

In this study, an NIR-responsive phase-transition MB (NPMB) system, which effectively treats cancer by controlling drug release through NIR irradiation, was designed based on the unique characteristics of MBs (Scheme 1). The system consists of an oil phase, 2H,3H-perfluoropentane (PFC), a fluorinated liquid–gas (vaporizes at a specific temperature), a photosensitizer, and an anticancer drug. In the absence of stimulation, IR-780 and paclitaxel (PTX) coexist in oil and PFC to maintain a stable MB state. IR-780 is a hydrophobic cyanine dye that effectively absorbs NIR light, which has a maximum absorption at approximately 780 nm, and converts it into photothermal energy to induce a local temperature increase [61,62,63]. PTX is a widely used hydrophobic anticancer drug that is effective against various types of cancer, including breast cancer, lung cancer, and ovarian cancer [64,65,66]. PFC undergoes a phase change from liquid to gas at a specific temperature, which induces MB expansion and collapses the MB structure. The oil phase increases the loading of IR-780 and PTX, which are hydrophobic substances, thereby facilitating efficient drug delivery in the body. Upon NIR irradiation, IR-780 generates heat via photothermal conversion, triggering the phase transition of the fluorinated gas, which causes MB collapse and subsequent drug release. The NPMB system developed in this study combines photothermal effects and bubble dynamics to enable precisely controlled drug release. This platform may help overcome nonspecific drug release and enable localized, targeted anticancer therapy with minimal side effects.

## 2. Materials and Methods

### 2.1. Materials

PTX (≥97%) and Doxorubicin (≥97%) were purchased from Ontario Chemicals, Inc. (Guelph, ON, Canada). Oleic acid was obtained from DAE JUNG (Gyeonggi, Republic of Korea). Glyceryl trioctanoate (≥99%, a medium-chain triglyceride (MCT), along with IR-780 iodide (≥95%), Pluronic F-127, phosphate-buffered saline (PBS), Dulbecco’s modified Eagle’s medium (DMEM), fetal bovine serum (FBS), and penicillin–streptomycin were purchased from Sigma-Aldrich (St. Louis, MO, USA). The compound 2H,3H-perfluoropentane (≥90%) was purchased from Thermo Scientific Chemicals (Waltham, MA, USA). HeLa cells were obtained from the Korean Cell Line Bank (Seoul, Republic of Korea). All the chemicals were used as received without further purification.

### 2.2. Instrumentation and Characterization

Fluorescence microscopy images were obtained using a confocal laser scanning microscope (CLSM) (TCS SP8, Leica Camera AG, Wetzlar, Germany). Absorption spectra were measured using a UV–Vis spectrophotometer (V-730, Jasco, Tokyo, Japan). Optical microscopy images were collected using a DM2700 M microscope (Leica Camera AG, Wetzlar, Germany). The hydrodynamic size distribution of microbubbles was determined using a ZEN3600 Dynamic Light Scattering system (Malvern Panalytical, Malvern, UK). NIR photostimulation was performed using an 808 nm continuous-wave diode laser system with an output power of 1.8 W/cm^2^ (Changchun New Industries Optoelectronics Technology Co., Ltd., Changchun, Jilin, China), and temperature changes during irradiation were recorded using a Ti95 infrared camera (Fluke, Everett, WA, USA). Cell viability was quantified with a microplate reader (Tecan Trading AG, Männedorf, Switzerland).

### 2.3. Preparation of Microbubbles (Blank MBs)

Oleic acid (100 μL) was added to a 30 mL vial. A mixture of MCT oil (150 μL) and 2H,3H-perfluoropentane (100 μL) was prepared separately and added to the oleic acid. The mixture was vortexed for 10 min and sonicated for 10 min in a bath sonicator. After sonication, the samples were placed on ice for 5 min. Pluronic F-127 (25 mg) was dissolved in 14 mL of PBS and added to the vial. The resulting mixture was vortexed for 10 s to disperse the MBs.

### 2.4. Preparation of IR-780-Loaded Microbubbles (IR-780@MB)

IR-780 (2.5 mg) was dissolved in chloroform and added to oleic acid (100 μL). After solvent evaporation under reduced pressure, a thin film was formed. A pre-mixed solution of MCT oil (150 μL) and 2H,3H-perfluoropentane (100 μL) was added to the film. The mixture was vortexed for 10 min, sonicated for 10 min in a bath sonicator, and stored on ice for 5 min. Pluronic F-127 (25 mg) was dissolved in 14 mL PBS and added to the mixture, followed by vortexing for 10 s to disperse the MBs.

### 2.5. Preparation of PTX-Loaded Microbubbles (PTX@MB)

PTX (2.5 mg) was dissolved in chloroform and added to oleic acid (100 μL). After solvent evaporation under reduced pressure, a thin film was obtained. A mixture of MCT oil (150 μL) and 2H,3H-perfluoropentane (100 μL) was added to the film, followed by vortexing and sonication under the same conditions as mentioned previously. Pluronic F-127 (stored on ice) was added to PBS, and the mixture was vortexed to produce PTX-loaded MBs.

### 2.6. Preparation of IR-780 and PTX Co-Loaded Microbubbles (NPMBs)

IR-780 (2.5 mg) and PTX (2.5 mg) were co-dissolved in chloroform and added to oleic acid (100 μL). After solvent evaporation, a thin film was formed. MCT oil (150 μL) and 2H,3H-perfluoropentane (100 μL) were added to the film. The mixture was vortexed for 10 min, sonicated for 10 min in a bath sonicator, and stored on ice for 5 min. Pluronic F-127 (25 mg) in 14 mL of PBS was added and vortexed for 10 s to obtain NIR-responsive phase-transition microbubbles (NPMBs).

### 2.7. Thermal Elevation Study

The thermal sensitivity of the NPMBs was evaluated by irradiating the samples with NIR light at different intensities (0.5, 0.7, 1.0, 1.2, and 1.8 W/cm^2^) for 10 min. Temperature changes were recorded using a thermal imaging camera. For the On–Off cycle test, NIR irradiation (1.8 W/cm^2^) was applied intermittently for 7 min intervals to monitor the thermal cycling behavior.

### 2.8. Thermal Stability Study

To investigate thermal stability, 1 mL of NPMB suspension was stored in vials and placed in a water bath set at 20 °C, 40 °C, 60 °C, and 80 °C for 1 h. Morphological changes were observed using optical microscopy after incubation.

### 2.9. Drug Release Study

To evaluate the NIR-triggered drug release behavior, NPMB suspensions were irradiated with NIR light at varying intensities (0.5–1.8 W/cm^2^) for 10 min using an 808 nm continuous-wave diode laser. After irradiation, the microbubbles, owing to their micrometer-scale size, naturally settled at the bottom of the vial. Without centrifugation, 1 mL aliquots of the supernatant containing the released paclitaxel (PTX) were carefully collected. A defined volume of the supernatant was mixed with methanol to ensure complete dissolution. The absorbance of the resulting solution was measured at 227 nm, the maximum absorption wavelength (λmax) of PTX, using a UV–Vis spectrophotometer. A standard calibration curve was constructed using PTX solutions of known concentrations, and the linear relationship between absorbance and concentration was used to determine the amount of released drug.

### 2.10. In Vitro Cell Viability Assay (MTT Assay)

HeLa cells were seeded in 96-well plates at a density of 3 × 10^3^ cells per well and incubated for 24 h in DMEM supplemented with 10% FBS and 1% penicillin–streptomycin at 37 °C and 5% CO_2_. Subsequently, cells were treated with supernatants collected after NIR-triggered drug release at different PTX concentrations (0.1–20 μM) and incubated for another 24 h. Cell viability was assessed employing the MTT assay: 20 μL of MTT solution (5 mg/mL) was added to each well, followed by 4 h of incubation. Formazan crystals were dissolved in DMSO, and the absorbance was measured at 570 nm using a microplate reader.

### 2.11. Live/Dead Cell Staining

HeLa cells were seeded at a density of 5 × 10^3^ cells per well in 12-well plates and cultured for 24 h. The cells were treated with the released drug supernatants under various conditions (with and without NIR irradiation). After treatment, cells were stained with fluorescein diacetate (FDA, 5 μM) and propidium iodide (PI, 2.5 μM) for 10 min at 37 °C. Live (green fluorescence) and dead (red fluorescence) cells were imaged using CLSM. Fluorescence intensities were quantitatively analyzed using the ImageJ software (version 1.53e, National Institutes of Health, Bethesda, MD, USA) [67].

## 3. Results and Discussion

To develop an MB system with controllable drug release in response to NIR irradiation, an oleic acid, PTX, and IR-780 solution was prepared, and the solvent was evaporated to produce a thin film. The F-127 surfactant, PFC, a fluorinated liquid/gas, and MCT oil, which increases the solubility of PFC in the system, were added to the film, and NPMB was subsequently formed through simple vortexing. Oleic acid shows low solubility in polar solvents but a strong affinity for hydrophobic drugs. Therefore, the solubility of the hydrophobic anticancer drug PTX and the thermosensitive IR-780 was increased. In particular, PTX has extremely low solubility in water, making it difficult to disperse it uniformly in a general aqueous environment; however, oleic acid facilitates the formation of a uniformly dispersed PTX film through hydrophobic interactions. However, because oleic acid has a low affinity for PFC, the additional use of MCT oil improved the formation and stability of MBs. This combination ensured the stability of the MBs, allowing them to maintain their size and shape and consequently providing a foundation for the precise control of drug release. PTX is a representative chemotherapeutic anticancer agent, and IR-780 is a photothermal material that specifically generates heat energy under NIR irradiation, which induces a phase transition of the PFC present inside the NPMB. Upon NIR exposure, IR-780 leads to a localized temperature increase in a short period of time, which causes the volume of gas inside the MB to expand and the internal pressure to rise simultaneously.

**Figure 1:**
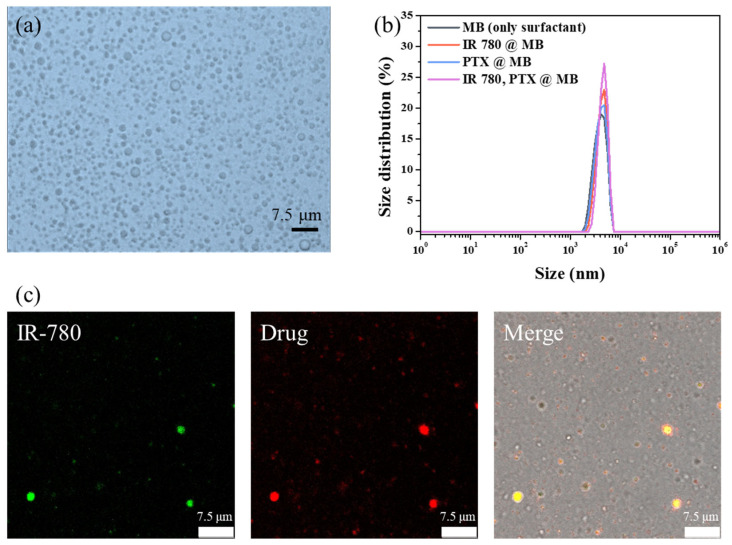
(**a**) Optical microscopy image of NPMBs. (**b**) Size distribution analysis of free MBs, IR-780-loaded MBs, PTX-loaded MBs, and NPMBs measured by DLS. (**c**) CLSM images of NPMBs showing green fluorescence from IR-780 and red fluorescence from the model drug.

This pressure increase eventually caused the collapse of the NPMB structure or rupture of its shell, which, in turn, immediately released the PTX encapsulated within the bubble. This characteristic significantly improves the precision and safety of the treatment by minimizing thermal damage to the surrounding healthy tissue and facilitating drug release exclusively from the target tissue. The synthesized NPMBs were characterized using optical microscopy, dynamic light scattering (DLS), and CLSM. Spherical particles were observed in the optical microscopy images (Figure 1a). In addition, the average sizes of free MBs, IR-780-loaded MBs, PTX-loaded MBs, and NPMBs were measured via DLS analysis, with all formulations exhibiting a diameter of approximately 4.2 μm, regardless of the presence or type of the loaded drug (Figure 1b). Furthermore, green fluorescence from IR-780 and red fluorescence from the model drug doxorubicin were observed. The overlap of both fluorescence signals within the NPMBs confirmed the successful co-loading of the two substances (Figure 1c). The hydrophobic form of doxorubicin was used as a model drug instead of PTX because PTX itself does not exhibit intrinsic fluorescence, making it unsuitable for fluorescence-based imaging.

**Figure 2:**
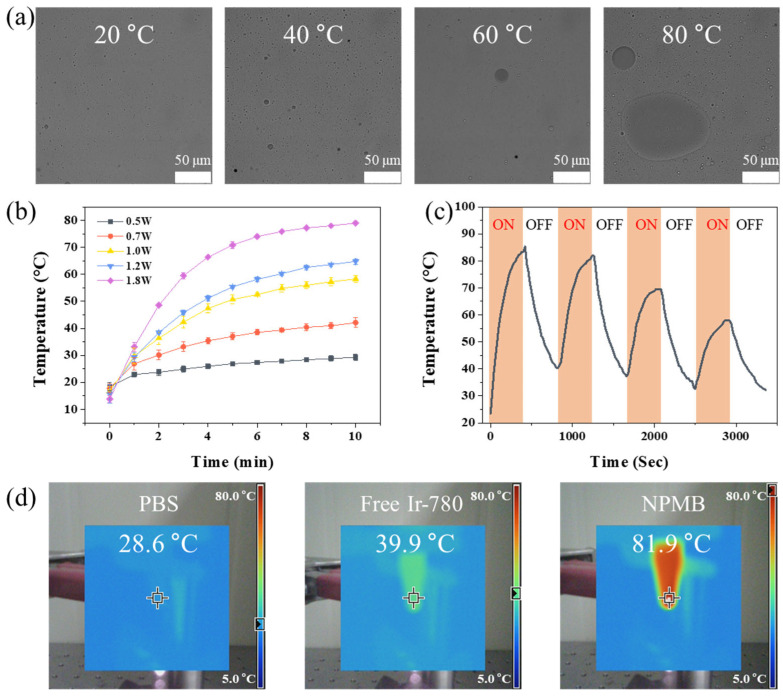
(**a**) Optical microscopy images showing morphological changes of NPMBs at various temperatures (20 °C, 40 °C, 60 °C, and 80 °C). (**b**) Temperature changes of NPMBs under different NIR irradiation intensities (0.5, 0.7, 1.0, 1.2, and 1.8 W/cm^2^). (**c**) Thermal response of NPMBs during repeated On–Off cycles of NIR irradiation. (**d**) Thermal imaging results comparing PBS solution, free IR-780, and NPMBs under NIR irradiation.

To evaluate the thermal sensitivity of NPMB, the morphological changes were observed under an optical microscope at various temperatures (20 °C, 40 °C, 60 °C, and 80 °C). The size of the NPMB particles gradually increased with rising temperature (Figure 2a). Furthermore, NIR-induced structural changes in the NPMBs, including expansion and partial collapse, were confirmed (Figure S1). This phenomenon is interpreted as the result of the volume expansion caused by the vaporization of the PFC inside NPMB, triggered by a phase transition occurring above the critical temperature (56 °C). The phase transition of the PFC immediately increased the internal pressure of the NPMBs and led to the rupture of their shell, destabilizing the structure and promoting the release of the encapsulated drug. The controllability of drug release via NIR irradiation was evaluated. NIR light was then applied at various intensities (0.5, 0.7, 1.0, 1.2, and 1.8 W/cm^2^), and the temperature of the NPMBs increased proportionally with irradiation intensity. When NIR was irradiated to NPMBs for 10 min at 0.5 W/cm^2^, the temperature rose to 29.3 °C, whereas, at 1.8 W/cm^2^, it increased to 79.0 °C (Figure 2b). This indicates that, as the NIR intensity increases, the amount of heat energy generated by IR-780 within the NPMBs also increases proportionally. The resulting temperature rise accelerates the phase transition of the PFC, inducing a more effective bubble collapse and facilitating drug release. In addition, a rise in temperature was observed with longer irradiation times; however, the maximum temperature was determined via the NIR radiation intensity rather than the duration of irradiation. To confirm that this phenomenon was caused by IR-780, NIR was irradiated on PBS solution, free IR-780, and NPMBs, and the temperature change was measured using a thermal imaging camera. No significant temperature increase was observed for the PBS solution. In free IR-780, the temperature increased to 39.9 °C upon NIR irradiation, which can be attributed to the poor dispersion and limited solubility of IR-780 in the aqueous environment. In contrast, NIR irradiation of NPMBs resulted in a marked temperature increase (81.9 °C), as confirmed through thermal imaging (Figure 2d). These results imply that NIR alone does not cause nonspecific heating and that heat is selectively generated only in MBs containing IR-780, supporting the targeting capability and biosafety of the system.

Significant thermal responses were observed during the On–Off cycles of the NIR irradiation. During the first irradiation, the temperature of NPMB rose to 89 °C and immediately dropped to 25 °C once the irradiation was turned off. The temperature increase tended to decrease with subsequent repeated irradiations. This phenomenon is interpreted as the result of vaporization of the PFC inside the NPMBs and precipitation of IR-780 following structural collapse, which gradually reduces the photothermal conversion efficiency. In the second, third, and fourth irradiation cycles, the maximum temperatures were observed to decrease to 85 °C, 70 °C, and 60 °C, respectively (Figure 2c). 

**Figure 3:**
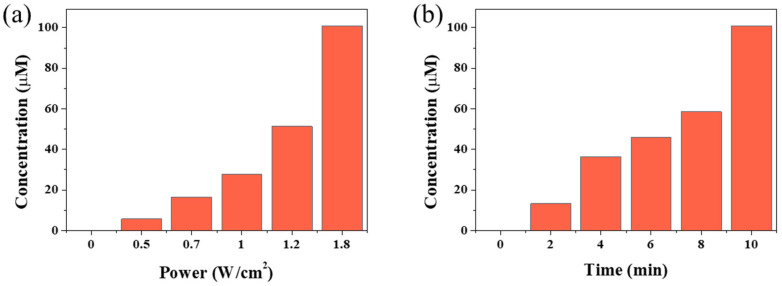
(**a**) Cumulative PTX release from NPMBs under different NIR irradiation intensities. (**b**) Time-dependent PTX release from NPMBs at 1.8 W/cm^2^.

The heat generated by NIR irradiation reduced the stability of NPMB, resulting in the release of the encapsulated PTX. To confirm the release of PTX, the amount released at different NIR intensities and irradiation times was measured. When irradiated at the highest intensity of 1.8 W/cm^2^, the amount of drug released was approximately 18 times greater than that released at 0.5 W/cm^2^ (Figure 3a). In addition, the amount of drug released continuously increased with irradiation times of 2, 4, 6, 8, and 10 min at 1.8 W/cm^2^, and approximately eight times more drug was released after 10 min of irradiation than after 2 min (Figure 3b). These results demonstrate that the NPMB system developed in this study can precisely control the timing and amount of drug released using only external stimulation (NIR), compared to conventional systems. 

**Figure 4:**
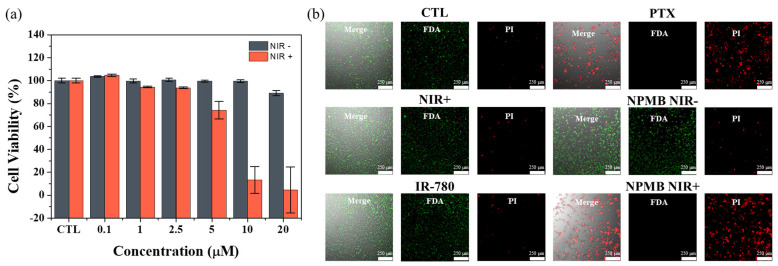
(**a**) Cell viability of HeLa cells treated with NPMBs under NIR irradiation. (**b**) Live/Dead cell staining images of HeLa cells under different treatment conditions: Control (untreated), NIR irradiation only, free IR-780, free PTX, NPMB without NIR irradiation, and NPMB with NIR irradiation. Green fluorescence (live cells, FDA staining) and red fluorescence (dead cells, PI staining).

To evaluate the therapeutic effect of the NPMB system, an NPMB encapsulating PTX and IR-780 was prepared, and drug release was induced through NIR irradiation. The supernatant containing the released drug was then incubated with the HeLa cell line, a human cervical cancer cell line, and cell viability was measured. Through NIR irradiation, the concentration of PTX released from NPMBs was adjusted from 0.1 μM to 20 μM, and cell viability was evaluated using the MTT assay. As a result, the cell viability decreased significantly to 4.6% at 20 μM, 13.4% at 10 μM, and over 74.2% at 5 μM or less. In contrast, under conditions without NIR irradiation, cell viability remained at 89.1% even at a maximum concentration of 20 μM, confirming that the drug release was effectively induced by NIR light (Figure 4a). These results imply that IR-780 induced photothermal conversion via NIR irradiation, which triggered the phase transition of the PFC, leading to the collapse of NPMBs and subsequent release of PTX, thereby inducing cytotoxicity. In contrast, in the absence of NIR irradiation, the NPMBs remained stable, preventing drug release and resulting in low cytotoxicity.

These results were confirmed using live/dead cell staining. The cells were divided into six groups for comparison: control (untreated), NIR irradiation only, free IR-780, free PTX, NPMB without NIR, and NPMB with NIR. Live cells were stained with green fluorescence using FDA, and dead cells were stained with red fluorescence using PI. In the control, NIR irradiation only, and IR780 only treatment groups, predominantly green fluorescent live cells were observed, while red fluorescence from dead cells was negligible. In the PTX-only treatment group, the number of red-stained dead cells increased moderately. In addition, in the NPMB-only treatment group (NIR non-irradiated), most cells retained their green fluorescence, indicating low cytotoxicity. In the NPMBs treatment group with NIR irradiation, the number of red-fluorescent dead cells significantly increased (Figure 4b). These results indicate that NIR irradiation induces the collapse of MBs and that PTX released during this process effectively triggers cancer cell death. Furthermore, these findings are consistent with the previous MTT assay results, suggesting that the NPMB system developed in this study could selectively release drugs and induce cancer cell death in response to NIR irradiation.

## 4. Conclusions

The NPMB system developed in this study demonstrated temporal and spatial control of drug release. The system selectively releases drugs at a desired time in response to external NIR stimulation, which is advantageous for minimizing unnecessary drug exposure outside the treatment area and reducing side effects.

In particular, IR-780-based photothermal conversion induced a sufficient temperature increase even with a short irradiation time and low laser power, facilitating effective drug release through the phase transition of the PFC and structural collapse of the MBs. This phase-transition-based collapse mechanism distinguishes the system from traditional ultrasound or single-function NIR-responsive carriers. The heat conversion efficiency was maintained after repeated NIR irradiation, indicating the thermal stability and potential reusability of the system.

The experimental results at the cellular level clearly demonstrated the selective drug release capability of this system. A significant concentration-dependent decrease in HeLa cell viability was observed solely under NIR irradiation, which triggered the release of PTX, whereas negligible cytotoxicity was observed in the absence of NIR irradiation. These findings emphasize that the system preserved structural stability and effectively suppressed drug release without NIR triggers. Furthermore, the Live/Dead cell staining results supported this finding, showing a significant increase in cell death only under NIR stimulation irradiation.

In conclusion, the NPMB system developed in this study facilitates precise control over the timing and amount of drug released in response to NIR stimuli and represents a promising drug delivery platform that combines photothermal effects and a bubble collapse mechanism for enhanced anticancer efficacy. With future validation of its in vivo efficacy and expansion to various drugs and therapeutic targets, this system holds strong potential as a next-generation light-responsive drug delivery technology. However, the current study is limited to in vitro validation. Further in vivo studies using appropriate animal models are necessary to confirm the clinical feasibility of the NPMB system and are planned as a future research direction.

## Data Availability

The original contributions presented in this study are included in the article and Supplementary Materials. Further inquiries can be directed to the corresponding author.

## Supplementary Materials

The following supporting information can be downloaded at: https://www.mdpi.com/article/10.3390/ma18122725/s1, Figure S1: Structural changes of NPMBs induced by NIR irradiation e; Figure S2: Cell viability analysis of individual components used in the NPMB; Figure S3: Quantitative analysis of red fluorescence intensity from PI-stained cells using ImageJ.

## Abbreviations

The following abbreviations are used in this manuscript:

MB	Micro bubble
NPMB	NIR-responsive phase-transition microbubble
NIR	Near infrared
PTX	Paclitaxel
CLSM	Confocal laser scanning microscopy
PFC	2H,3H-perfluoropentane
MCT	Medium-chain triglyceride
FDA	Fluorescein diacetate
PI	Propidium iodide
