# Supplementary material for: NIR-Responsive Microbubble Delivery Platforms for Controlled Drug Release in Cancer Therapy
Source: Materials (Basel). 2025 Jun 10;18(12):2725. doi: 10.3390/ma18122725 (PMC12194853; doi:10.3390/ma18122725)
Supplement: Supplementary file 1 [file materials-18-02725-s001.zip › materials-3659336-supplementary.pdf]

Supporting information for:

NIR-Responsive Microbubble Delivery Platforms for Controlled Drug Release in Cancer Therapy

Kibeom Kim [1,2 †], Been Yoon [3 †], Jungmin Lee [3], Gyuri Kim [1] and Myoung-Hwan Park[1,2,3 *]

1 Department of Convergence Science, Sahmyook University, Seoul 01795, South Korea; kibumsy@syu.ac.kr (K.K.); gyurikim716@gmail.com (G.K.)
2 Department of Chemistry and Life Science, Sahmyook University, Seoul 01795, Republic of Korea
3 N to B Co., Ltd., Business Incubator Center, Hwarang-ro, Nowon-gu, Seoul 01795, Republic of Korea; beeny0102@gmail.com (B.Y.); jm.lee090394@gmail.com (J.L.)
* Correspondence: mpark@syu.ac.kr
† These authors contributed equally to the work.

Figure S1. **Structural changes of NPMBs induced by NIR irradiation**

Figure S2. **Cell viability analysis of individual components used in the NPMB**

Figure S3. **Quantitative analysis of red fluorescence intensity from PI-stained cells using ImageJ**

Structural changes of NPMBs induced by NIR irradiation

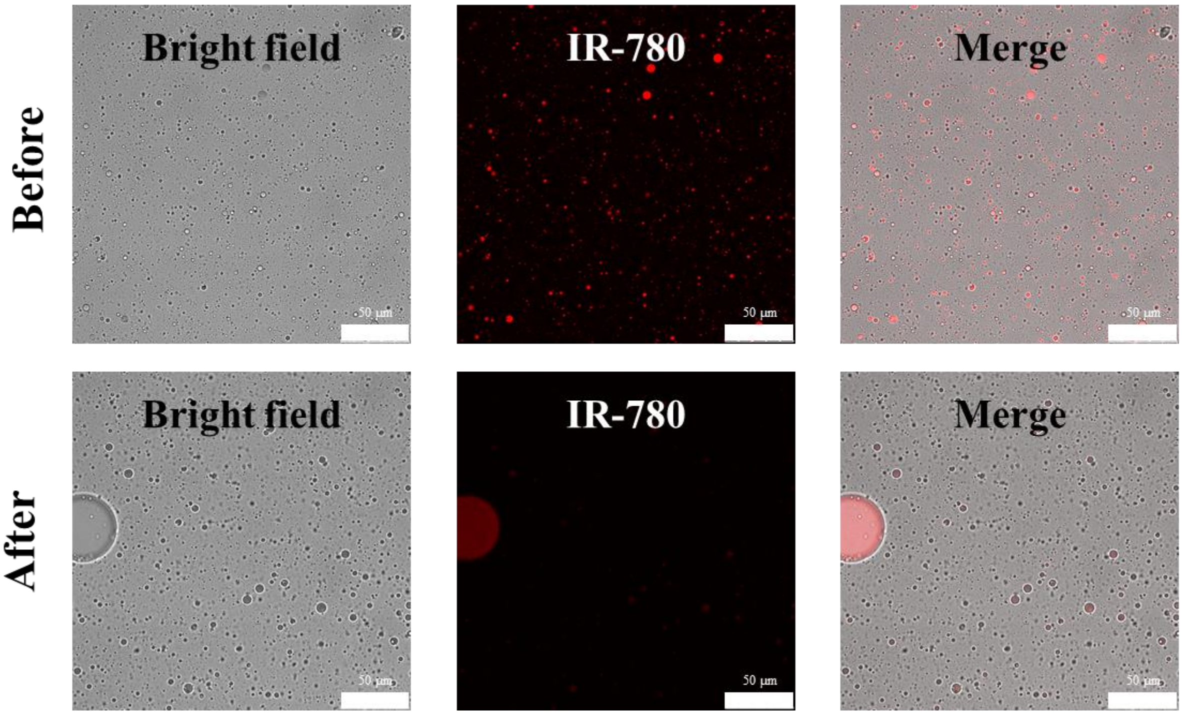

Figure S1. Bright-field, fluorescence (IR-780), and merged images of NPMBs before (top) and after (bottom) NIR irradiation (1.8 W/cm², 10 min). After irradiation, a significant increase in microbubble size and fluorescence intensity is observed, indicating photothermal-triggered bubble expansion and drug release. Scale bar = 50 µm.

Cell viability analysis of individual components used in the NPMB

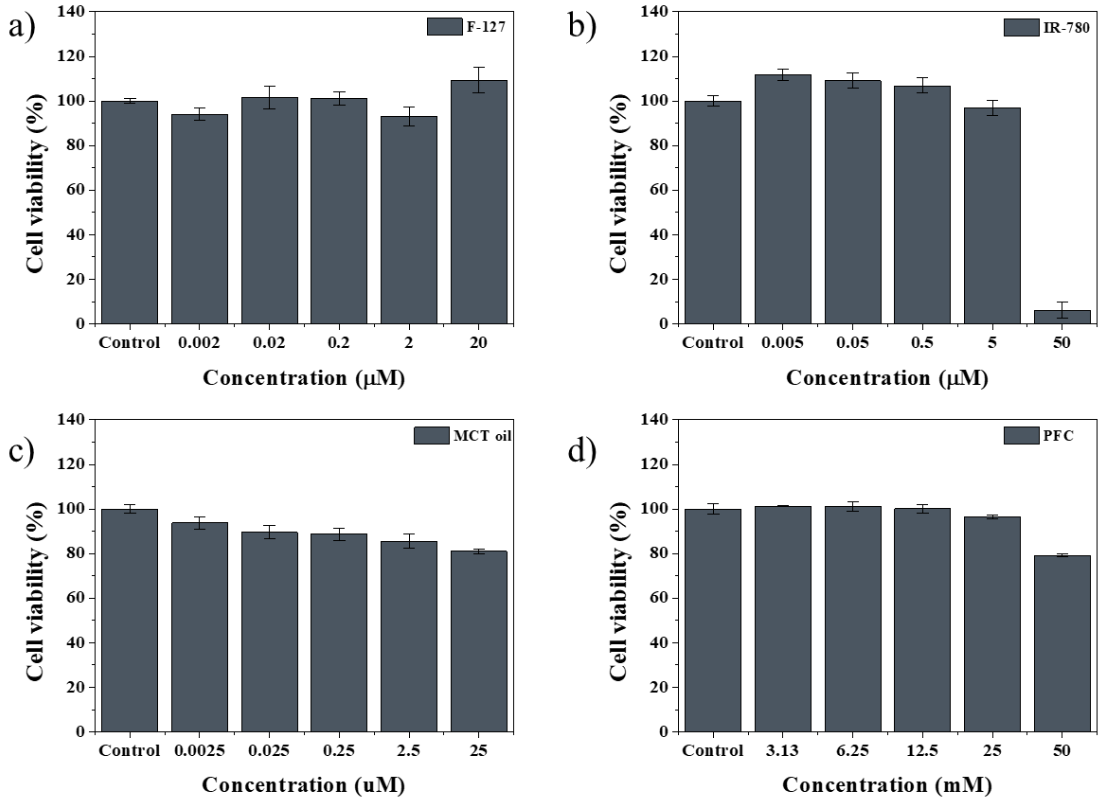

Figure S2. Cell viability (%) of HeLa cells after 24-hour exposure to varying concentrations of (a) Pluronic F-127, (b) IR-780, (c) MCT oil, and (d) PFC assessed by MTT assay. F-127 and MCT oil exhibited minimal cytotoxicity across the tested concentration ranges. IR-780 showed dose-dependent cytotoxicity, with cell viability significantly decreasing at 50 μM. PFC showed no noticeable cytotoxicity even at the highest tested concentration (50 mM), confirming its high biocompatibility. Data are presented as mean ± SD (n = 3).

Relative red fluorescence intensity of PI-stained cells across treatment groups

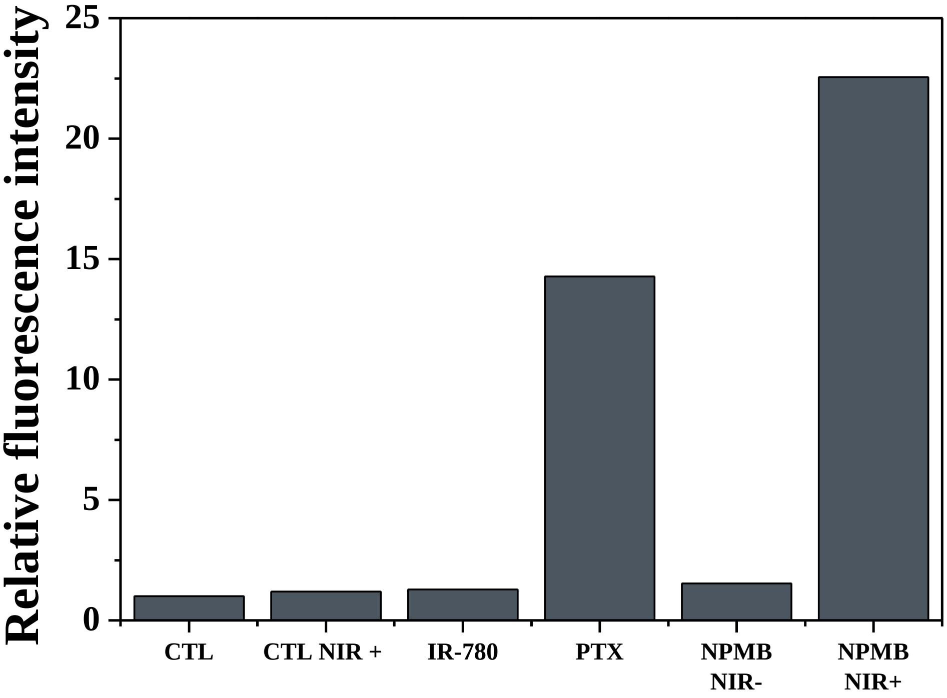

Figure S3. Relative red fluorescence intensity of propidium iodide (PI)-stained HeLa cells under various treatment conditions. Fluorescence values were quantified using ImageJ from images obtained after FDA/PI co-staining. Data were normalized to the control group to highlight the extent of NIR-triggered cytotoxicity. The NPMB + NIR group showed a markedly increased fluorescence signal compared to other groups, indicating enhanced cell death due to triggered drug release.
